# Advanced Myoelectric Control for Robotic Hand-Assisted Training: Outcome from a Stroke Patient

**DOI:** 10.3389/fneur.2017.00107

**Published:** 2017-03-20

**Authors:** Zhiyuan Lu, Kai-yu Tong, Henry Shin, Sheng Li, Ping Zhou

**Affiliations:** ^1^Department of Physical Medicine and Rehabilitation, University of Texas Health Science Center at Houston, TIRR Memorial Hermann Research Center, Houston, TX, USA; ^2^Division of Biomedical Engineering, Department of Electronic Engineering, The Chinese University of Hong Kong, Hong Kong, Hong Kong; ^3^Guangdong Work Injury Rehabilitation Center, Guangzhou, China

**Keywords:** electromyography, myoelectric pattern recognition, hand exoskeleton, rehabilitation, case report

## Abstract

A hand exoskeleton driven by myoelectric pattern recognition was designed for stroke rehabilitation. It detects and recognizes the user’s motion intent based on electromyography (EMG) signals, and then helps the user to accomplish hand motions in real time. The hand exoskeleton can perform six kinds of motions, including the whole hand closing/opening, tripod pinch/opening, and the “gun” sign/opening. A 52-year-old woman, 8 months after stroke, made 20× 2-h visits over 10 weeks to participate in robot-assisted hand training. Though she was unable to move her fingers on her right hand before the training, EMG activities could be detected on her right forearm. In each visit, she took 4× 10-min robot-assisted training sessions, in which she repeated the aforementioned six motion patterns assisted by our intent-driven hand exoskeleton. After the training, her grip force increased from 1.5 to 2.7 kg, her pinch force increased from 1.5 to 2.5 kg, her score of Box and Block test increased from 3 to 7, her score of Fugl–Meyer (Part C) increased from 0 to 7, and her hand function increased from Stage 1 to Stage 2 in Chedoke–McMaster assessment. The results demonstrate the feasibility of robot-assisted training driven by myoelectric pattern recognition after stroke.

## Introduction

Robot-assisted upper limb training is considered to be more efficient (1) and economic (2) than conventional therapy in neurorehabilitation. Controlling the robot with the user’s own electromyography (EMG) signals connects the user’s intended motion and his actual movements. It can therefore enhance therapeutic effects and promote motor learning (3–5). Various EMG-driven robots and exoskeletons have been developed for neurorehabilitation (6–8), primarily based on one-to-one mapping, which typically maps one channel of EMG signal to a corresponding single degree-of-freedom (DOF) or variable such as speed and torque using a conventional “on-off” or proportional strategy. Robots based on such control strategy work well on training joints with only a few DOFs such as elbow and wrist. However, a human hand has up to 27 DOFs (9) and is controlled by complex temporal and spatial coordination of multiple muscles. It is therefore not feasible to regain hand dexterity through conventional control strategies. Myoelectric pattern-recognition techniques have been developed to extract motion intentions from EMG signals (10, 11). The extracted intentions can then be used to control a multiple-DOF robot such as a prosthesis (12). Previous studies have also shown that motion intentions can still be extracted after neurological impairment (13–15). We therefore developed an intent-driven hand training system. The system employs an exoskeleton hand, which is controlled by myoelectric pattern recognition. As soon as the user’s intention is detected (usually within 250 ms), the system is able to assist to accomplish the intended motions (16).

## Case Report

### Subject

A 52-year-old woman participated in this robotic hand-assisted training 8 months after stroke. She was right-handed before stroke and had hemiplegia on her right side after her stroke. She was able to walk independently with an ankle foot orthosis but had difficulties in moving her right arm. Her fingers were flexed naturally. She was unable to move any of the fingers on her right hand, but EMG signals were able to be recorded from her forearm. Her Fugl–Meyer score (Part A–D, max 66) was 16, with a 0 in Part C (Hand, max 14). She had no pain when her whole hand was passively opened or closed. She did not receive any other hand or upper limb therapies while participating in this study. During her visits, she was able to understand and follow all the instructions.

### Exoskeleton Hand

**Figure 1:**
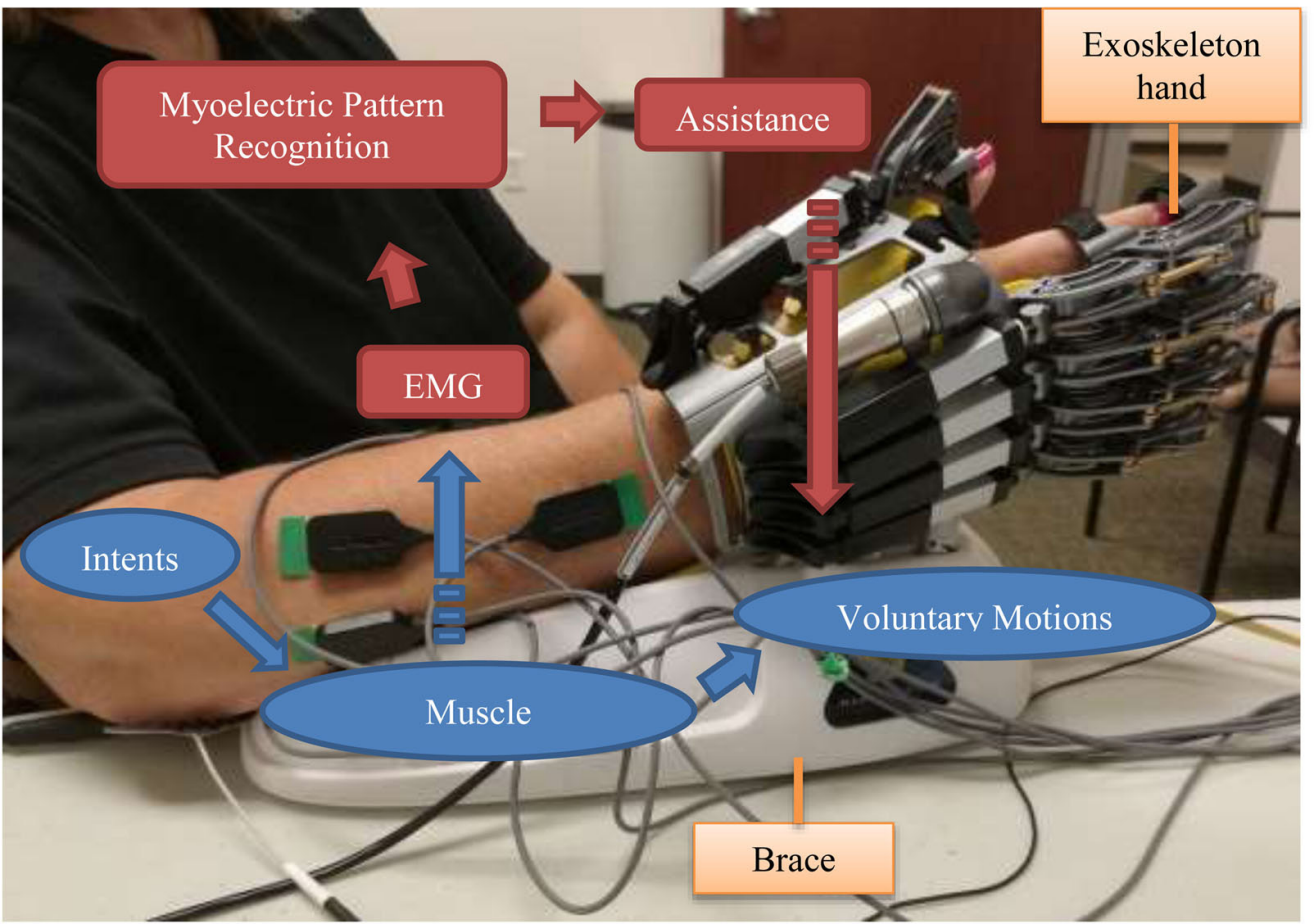
**Training with the exoskeleton hand driven by myoelectric pattern recognition**.

The exoskeleton hand, Hand of Hope (Rehab-Robotics, Hong Kong), was used in this study to help the subject move her hand (Figure 1). The exoskeleton hand has five individual fingers. Each finger is actuated by a linear actuator that can pull and push linearly. The mechanical design of the fingers converts these linear movements into the rotations of a virtual metacarpophalangeal (MCP) joint and a virtual proximal interphalangeal (PIP) joint. Both joints rotate together to help the hand perform closing and opening movements (7). The motion range is 55° and 65° for MCP and PIP joints, respectively. The subject’s palm and five fingers are fixed to the exoskeleton hand with Velcro belts. Each finger can be bent or straightened individually by the exoskeleton hand. The exoskeleton hand stands on a brace, which also supports the subject’s forearm, so that the subject can be totally relaxed when attached to the exoskeleton. The exoskeleton hand used in this study can perform six different motion patterns, including hand closing (HC); hand opening (HO); thumb, index, and middle fingers closing (TIMC or tripod pinch); thumb, index, and middle fingers opening; middle, ring, and little fingers closing (MRLC or the “gun” sign); and middle, ring, and little fingers opening. The exoskeleton hand can perform HC, TIMC, or MRLC when it is open. However, after performing any one from these three patterns, it can only return to the original open status (e.g., there is no direct way from the “tripod pinch” to the “gun” sign).

Conventional EMG control of the device was applied in previous studies for training hand opening/closing function for stroke survivors (17, 18). In order to make all these six motion patterns available for the subject, a myoelectric pattern-recognition system was developed for this study to control the exoskeleton hand. This system is able to detect and recognize the subject’s muscle activity patterns, indicate his/her intended hand motions, and then assist the patient in accomplishing these motions in real time. When the subject tries to perform a hand motion, EMG signals can be detected from those activated muscles. The myoelectric pattern-recognition system then extracts the motion intent from these EMG signals and maps the intent into control commands. The exoskeleton hand therefore performs the same motion as the subject’s intent, so that the subject can accomplish the motion with both robotic assistance and his/her own participation.

### Protocol

The subject made 20 visits (experiments) for the robot-assisted training, 2 visits per week. During the experiment, the subject was seated comfortably in a chair, next to a small height-adjustable side table. The exoskeleton hand was placed beside her on the table on her right side. Her right hand was fixed in the exoskeleton, and her forearm was placed on the brace (Figure 1). The exoskeleton was placed and locked on the brace. Therefore, the subject’s right arm and hand could be totally relaxed instead of resisting gravity. The height of the table was adjusted to make the angle between her upper arm and her trunk about 45°, and the angle between her upper arm and her forearm about 90°. The subject was free to move her left hand. She was also allowed to move her right arm by moving the brace when she took breaks between two training sessions. Considering that the virtual palm was locked, the subject’s right hand was always in a neutral position, and her forearm was never rotated even when she moved the brace. The brace would be moved to its initial position before another training session began.

Seven bipolar surface electrodes (Delsys 2.1) were attached on the subject’s forearm using double-sided tapes, covering the first dorsal interossei, flexor digitorum superficialis, flexor digitorum profundus, extensor digitorum, abductor pollicis longus, extensor digiti minimi, and extensor pollicis longus muscles. The reference electrode was placed on the olecranon. The skin was cleaned using sterile alcohol wipes before electrodes were placed. EMG signals were acquired using a Bagnoli-8 EMG System (Delsys Inc., Boston, MA, USA), which amplified raw EMG signals 10,000 times and filtered the signals using a 20–450 Hz band pass filter. The acquired EMG signals were then input into a desktop running Windows 7 through a data acquisition device, USB-6221 (National Instruments Inc., Austin, TX, USA), which digitized the signals at 1,000 Hz with a 16-bit resolution.

The digitized EMG signals were then analyzed by a myoelectric pattern-recognition program developed for this study, which aimed to extract motion intentions from multi-channel EMG signals. The analysis was based on the EMG signals recorded in the most recent 200 ms (named a processing window) and was performed every 100 ms, so that the recognition result could be updated at 10 Hz (19), which is acceptable for real-time control. A motion detection algorithm was first performed to tell whether this window contained EMG signals that corresponded to the user’s voluntary motions. It calculated the mean absolute value (MAV) (20) of the processing window. If the MAV was smaller than a given threshold (it was 80% of the average MAV of EMG signals recorded from this subject at her median force level), no further processing would be performed, and the recognition result was “no motion.” Otherwise, a motion was considered to be detected because one or more muscles were active. Then, a pattern-recognition algorithm was performed, in which a support vector machine classifier (21) was applied to recognize motions based on a set of features including root mean square amplitude (20), fourth order auto regressive coefficients (22), and waveform length (20). The output of the classifier was then mapped onto control commands and sent to the hand exoskeleton.

Each visit included five sessions. In the first session, the subject repeated each motion pattern 15 times. Although she was unable to move her fingers, she was encouraged to try controlling the fingers. For each motion pattern, the subject was asked to imagine moving her fingers in the desired motion. The first 2 s of above-threshold EMG signals were recorded before the exoskeleton subsequently provided assistance corresponding to the motion pattern. These EMG signals recorded in these 2-s periods were used to train the classifier. The next four sessions were all training sessions. In each session, the subject controlled the exoskeleton using her own motion intent. She was asked to try performing all of the six motion patterns and then to follow through with the exoskeleton hand while it moved through its full motion range. When the exoskeleton reached the final range, it stopped, and began waiting for another motion intent from the subject. The subject was therefore encouraged to perform the next motion right after the exoskeleton stopped. The subject was always free to choose any of the executable patterns, but sometimes the experimenter gave suggestions in order to balance the training amount of each motion pattern. Considering the subject’s EMG signals were very weak, it was necessary for her to perform motions using about 70% of her maximal force in all these sessions. Consequently, she got fatigued quickly after 8–10 min training. In order to avoid fatigue, each training session was set to 10 min and could be terminated at any time after 8 min. Moreover, she was given as much time as needed to rest between sessions. Therefore, each visit took about 2 h, including approximately 40 min training.

## Results

Assessments, including grip force (using Jamar Plus + Digital Hand Dynamometer, Patterson Medical, Warrenville, IL, USA), pinch force (using PG-60 Pinch Gauges, B&L Engineering, Santa Ana, CA, USA), Box and Block test (23), Fugl–Meyer (Part C only, ranging from 0 to 14), and Chedoke–McMaster (the Hand Stage only, ranging from 1 to 7) (24), were performed before and after the 20-visit training. Results are shown in Table 1. The results of both the grip force and the pinch force were the maximal readings from three measurements. The result of Box and Block test was the highest score of three trials. As to the Fugl–Meyer and Chedoke–McMaster assessment, only the hand-related score were reported because only hand function was trained. The average control accuracy of the 1st and the 20th visit was also calculated. The subject was requested to report every time when the exoskeleton hand performed a motion that was different from her intent. The control accuracy was calculated based on the number of wrong motions and the total number of motions.

**Table 1:** **Assessment results before and after the training**.

Tests	Pretreatment	Posttreatment
Grip force (kg)	1.5	2.7
Pinch force (kg)	1.5	2.5
Box and Block	3	7
Fugl–Meyer (part C)	0	7
Chedoke–McMaster (hand)	1	2
Control accuracy (%)	75.0	76.9

After the training, the results of all the assessments were improved dramatically. The grip force and pinch force were almost doubled. She also regained some voluntary finger movements. These improvements were quantified by three functional assessments from different aspects. Before the training, she was not able to perform observable finger movements. Her Fugl–Meyer score (Part C) was 0 and Chedoke–McMaster stage was 1 because she failed to do all the tasks in these assessments. After the training, she could flex all her fingers in a small range, so that she was able to partially perform many of the tasks. For example, she was able to hold a pencil, though loosely. As a result, she obtained 1 point in each grip task in the part C of the Fugl–Meyer assessment except the task “flexion in interphalangeal joints and extension in MCP.” Also because of these active motions, she met the criteria of Hand Stage 2 in the Chedoke–McMaster assessment. However, she did not get to Stage 3 because her range of motion was not greater than 50% plus that she did not have opposition to bring the thumb to the index finger. The same functional improvement increased her score in the Box and Block test. Because she was not able to open/close her hand before the training, she developed an alternative way to accomplish this task, which was to push a block into the space between her thumb and other fingers using arm movements. When she managed to push two corners of a block into her hand, she could pick up the block. Holding the block in hand was difficult given that her grip was weak and she was not holding the whole block. As a result, preventing dropping the block half way was more challenging for her, compared with releasing the block. After the training, she used the same way to move the blocks. Although the range of motion for HO was still not large enough to pick up or hold one block, it was easier for her to push the block in. And she could hold the block for a longer time because of her increased grip force, so that she had more time to move the block over the barrier. The scores of her three trials after training were 4, 7, and 4, respectively, while her highest score before training was 3.

## Discussion

In the previous studies applying conventional control (17, 18), the exoskeleton was triggered when the EMG amplitude of the monitored muscle(s) exceeded a given threshold, while other muscles’ activities were ignored. However, finger motions are generated by coordinating a series of muscles. Pattern-recognition algorithms were therefore introduced in this study in order to analyze the motion patterns of up to seven muscles. These algorithms also made it possible to control the hand exoskeleton in multiple DOFs and provided an approach to training fine motions and improving hand dexterity. Both the myoelectric pattern-recognition techniques and the robot-assisted training are safe. No adverse event was observed, and no discomfort was reported. Although fatigue was reported sometimes, it usually went away after a few minutes break.

This intent-driven control required the subject to be active during the training. This subject was able to activate her muscles though she could not perform finger motions. The real-time assistance from the exoskeleton gave her the feedback of muscle activities and helped her strengthen her motion patterns. Although the subject had severely impaired hand functions before the training (Hand Stage 1 according to the Chedoke–McMaster assessment), she still achieved 75% control accuracy. Our algorithms recognized most of the subject’s motion intents correctly, which assisted her in accomplishing these motions. Her hand function improved after the training, and all the assessments showed consistent improvements.

Although the training program for this subject demonstrates promising outcomes, a comprehensive evaluation of the effectiveness of the robotic hand-assisted training driven by myoelectric pattern recognition requires testing with a larger number of stroke subjects. We are aware that for a wide range of stroke patients with mild to severe impairment, some patients may not be suitable for such training due to lack of muscle activity or impaired muscle activity patterns (25, 26), while those stroke subjects who are able to generate muscle activity patterns and achieve reasonable accuracies can participate in the training program. In this regard, a pre-examination or assessment might be necessary to determine the stroke subjects who are able to control the exoskeleton hand with myoelectric pattern recognition, and who can benefit most from the robotic hand aided training.

## Ethics Statement

This study was approved by the Committee for the Protection of Human Subjects (CPHS) of The University of Texas Health Science Center at Houston and TIRR Memorial Hermann (Houston, TX, USA). All procedures of the study were performed in accordance with the Declaration of Helsinki. The subject gave written and informed consent before the experimental procedures.

## Author Contributions

ZL performed experiment, analyzed data, and wrote the manuscript. KT developed the robotic exoskeleton hand and helped with data analysis. HS helped with experiment and data analysis. SL helped with data analysis and interpretation. PZ oversaw the study and helped with experiment, data analysis, and interpretation. All the authors read, revised, and approved the manuscript.

## Conflict of Interest Statement

The authors declare that the research was conducted in the absence of any commercial or financial relationships that could be construed as a potential conflict of interest.
